# Pay attention to the basal ganglia: a volumetric study in early dementia with Lewy bodies

**DOI:** 10.1186/s13195-019-0568-y

**Published:** 2019-12-21

**Authors:** Anne Botzung, Nathalie Philippi, Vincent Noblet, Paulo Loureiro de Sousa, Frédéric Blanc

**Affiliations:** 1 Geriatrics and Neurology Departments, Research and Resources Memory Center (CM2R), Strasbourg University Hospital, Strasbourg, France; 2 ICube laboratory (CNRS, UMR 7357) and FMTS (Fédération de Médecine Translationnelle de Strasbourg), University of Strasbourg, Strasbourg, France

**Keywords:** Dementia with Lewy bodies, Prodromal, Mild cognitive impairment, Attention, Processing speed, Neuroimaging, MRI, VBM, Basal ganglia

## Abstract

**Background:**

Cortical and subcortical cognitive impairments are usually found in dementia with Lewy bodies (DLB). Roughly, they comprise visuo-constructive/executive function and attention/processing speed impairments, whereas memory would remain relatively spared. In this study, we focused on the neuro-anatomical substrates of attention and processing speed, which is still poorly understood. For the purpose of the study, we examined the correlations between behavioral scores measuring the speed of processing and the degree of cerebral atrophy in patients with prodromal to moderate DLB.

**Methods:**

Ninety-three prodromal to moderate DLB patients (mean MMSE = 25.5) were selected to participate in the study as well as 28 healthy elderly subjects (mean MMSE = 28.9), matched in terms of age and educational level. The Trail Making Test A (TMTA) and the Digit Symbol Substitution Test (DSST) were used to assess attention and processing speed. Behavioral performances were compared between patients and healthy control subjects. Three-dimensional MRI images were acquired for all participants, and correlational analyses were performed in the patient group using voxel-based morphometry (VBM).

**Results:**

The behavioral results on both the TMTA (*p* = .026) and the DSST (*p* < .001) showed significantly impaired performances in patients in comparison with control subjects. In addition, correlational analyses using VBM revealed for the TMTA negative correlations in the caudate nucleus (left cluster peak significant at .05 FWE corrected), the putamen, the left thalamus, and the subthalamic nuclei (*p* < .05 FDR corrected). Some positive correlations associated with the DSST were found in the right inferior frontal gyrus, the left thalamus, and the left cerebellum (*p* < .001 uncorrected).

**Conclusions:**

The behavioral results are in line with the literature on the DLB cognitive profile and confirm the existence of attention and processing speed impairment. Interestingly, VBM analysis revealed the involvement of the basal ganglia, in particular, the left caudate nucleus, which is part of the attention cerebral network, suggesting an important role of this structure for attentional processing speed. This also suggests the clinical implication of damage in this region relatively early in the course of the disease.

## Background

Dementia with Lewy bodies (DLB) is the second most common form of neurodegenerative disease after Alzheimer’s disease (AD), with prevalence rates up to 5% in the elderly population and up to around 20% of all cases of dementia [1, 2]. According to the most recent consensus criteria [3], a diagnosis of probable DLB can be made if two or more core clinical features are present, among fluctuating cognition, recurrent visual hallucinations, rapid eye movement sleep behavior disorder (RBD), and spontaneous features of parkinsonism. But essential to the diagnosis of DLB remains the progressive cognitive decline, with deficits on tests of attention, executive functions, and visuo-perception in the foreground, which may occur early [4, 5]. Among these deficits, attentional dysfunction, which is typically assessed in clinical routine by measures of speed of processing, is a prominent and distinguishing neuropsychological feature of DLB as compared to AD at the prodromal stages [4, 6]. For instance, based upon a large prospective study comparing DLB with AD, the slowing of cognitive processing (i.e., deficits of cognitive reaction time) appears to be specific to DLB in the early stages of the disease [7]. Tasks of attention and processing speed requiring a graphomotor response seem to be particularly useful in discriminating DLB from normal status or versus AD [6]. Previous studies have also indicated a disruption of attention in the visual modality in DLB as compared to AD and Parkinson’s disease (PD) patients [8–11].

Attention is a broad concept and, accordingly, many supporting brain regions are involved. Current neuroanatomical descriptions mostly come from the literature on fMRI in normal subjects and are drawn in a network perspective: in the context of attention-consuming tasks, the dorsal attention network (DAN) and the salience network (SN) both exhibit increased activity whereas the default network (DN), including regions of the cortical midline, is disengaged [12, 13]. The DAN encompasses the frontal eye field and inferior parietal sulcus and is involved in directed attention and working memory (Corbetta and Shulman [14]). The SN involves the anterior insula (AI), which plays a prominent role in the detection of salient stimuli, and the dorsal anterior cingulate cortex (dACC), more involved in task control [15]. The subcortical areas, including the thalamus, striatum, superior colliculus, and connecting white matter tracts, also participate in these networks [16].

Interestingly, structural neuroimaging studies using voxel-based morphometry (VBM) in patients with DLB, relative to AD patients, PD patients, and/or normal controls, have revealed gray matter (GM) atrophy in some of the abovementioned attentional regions. If relative to AD patients, DLB patients show a relative preservation of medial temporal lobe volumes [17–20]; compiling VBM studies of DLB patients versus control subjects revealed consistent decreased GM volumes in the right lateral temporal/insular cortex and left lenticular nucleus (putamen and globus pallidus)/insular cortex [21]. These data are at least partially corroborated by studies conducted in our team with DLB patients versus controls, showing reduced GM volumes of the bilateral insula and anterior cingulate cortex in prodromal DLB (pro-DLB) using VBM [22], reduced GM volumes of the bilateral insula in pro-DLB using DARTEL-VBM, and diminished right insula and right orbito-frontal cortex volumes in pro-DLB using measures of cortical thinning [23]. Additionally, reduction of GM density has been shown in the occipito-parietal areas and bilateral lenticular nucleus in DLB versus PD [24], in the left caudate nucleus in DLB versus controls [25], in the bilateral thalamus in DLB versus controls [26], and in the bilateral putamen in DLB versus controls and AD [27].

So far, the question of the cerebral regions involved in attention deficits in DLB patients has been investigated in a few papers only. In an fMRI study, Firbank at al [28]. have shown in Lewy body disease (LBD) patients (DLB and PD) greater activation of the DAN for incongruent and more difficult trials as well as heightened deactivation of the default network, interpreted as an attempt to allocate resources to impaired attentional networks. Using functional connectivity analysis in the same cohort of patients, Koboleva et al. [29] found a decreased connectivity between the DAN and the ventral regions. Based on the same cohort again, Cromarty et al. [30], in a VBM study, did not show any significant correlations between attentional performance, despite deficits in the tasks, and GM volumes, suggesting, according to the authors, that these effects were unlikely due to region-specific structural deficits. On the other hand, Watson et al. [26] found in a pure group of DLB patients that impaired attentional function (measured by simple and choice reaction times) was correlated to the left thalamic regions (pulvinar and ventral lateral nucleus). Overall, these studies have not so far provided a clear picture of the regions involved in the attentional deficits of DLB patients.

The aim of the present study was to better understand the underlying structural mechanisms of the attentional deficit in DLB patients. Based on the fact that both the insular cortex and basal ganglia (caudate and thalamus) are important to attention function, that both areas are highly interconnected [31, 32], and given that these regions are atrophied at an early stage in DLB patients, we posit that attention disturbances in DLB might be related to diminished volume in these regions. To test this hypothesis, we took advantage of a unique cohort of 93 patients diagnosed with prodromal to moderate DLB, who underwent a full neuropsychological assessment, including attentional measures, as well as structural MRI.

## Methods

### Participants

Ninety-three prodromal to moderate DLB patients were selected to participate in the study as well as 28 healthy elderly control subjects (HCs), matched in terms of age and educational level (see Table 1). Patients were recruited from the tertiary memory clinic of Strasbourg University Hospital, France, including the geriatrics and neurology departments. HCs were recruited among friends and relatives of the patients or via the listing of controls of the local clinical investigation center. In all patients, the diagnosis was made by clinicians with expertise in dementia, who performed a complete anamnesis and medical and neurologic examination, encompassing the 4 core features of McKeith et al.’s criteria [3]. Features of parkinsonism were evaluated using the Unified Parkinson’s Disease Rating Scale (UPDRS, part 3): akinesia, rigidity, and tremor at rest (rated from 0 for no symptoms to 4 for serious symptoms). Fluctuations were assessed with the Mayo Clinic Fluctuations scale [33] and the Newcastle upon Tyne Clinician Assessment of Fluctuation scale [34]. The Parkinson’s disease-associated psychotic symptoms questionnaire [35] was used to evaluate the presence of hallucinations. RBD was evaluated using a sleep questionnaire on RBD from the publication by Gjerstad et al. [36], simplified into 4 questions for the patient and the caregiver: one concerning movements during sleep, the second concerning vivid dreams and nightmares. Patients with prodromal DLB were defined as patients with MCI and preservation of independence (assessed by the Instrumental Activities of Daily Living [37, 38], DSM-5 [39], and McKeith’s criteria [1, 3] meeting probable DLB criteria except for the presence of dementia).

**Table 1:** Demographic and clinical characteristics of dementia with Lewy bodies (DLB) patients and healthy control subjects

Characteristics	DLB patients (*N* = 93)	Controls (*N* = 28)	*p/χ*^2^ value
Age, years	70.3 (9.7)	67.6 (7.8)	*p* = .189
Education, years	11.3 (4.1)	12.9 (2.7)	*p* = .063
Sex, M/F	52/41	16/12	*χ*^2^ = .908
Handedness, R/L	83/10	26/2	*χ*^*2*^ = .575
Fluctuations (%)	49/93 (52.7)	1/28	*χ*^*2*^ *< .001*
Hallucinations^a^ (%)	56/91 (61.5)	3/26	*χ*^*2*^ *< .001*
Parkinsonism			
Akinesia (%)^b^	58/90 (64.4)	0/28	*χ*^*2*^ *< .001*
Rigidity (%)^b^	60/90 (66.7)	0/28	*χ*^*2*^ *< .001*
Tremor at rest (%)^c^	17/85 (20)	2/28	*χ*^2^ = .115

Standard deviations for age and education are shown in parentheses. Significant *p* and *χ*^2^ values are in italics

^a^Data missing for two patients

^b^Data missing for three patients and two controls

^c^Data missing for eight patients

Exclusion criteria for all participants included a history of alcohol/substance abuse, evidence suggesting alternative neurological or psychiatric explanations for symptoms/cognitive impairment (for patients), or the presence of other severe or unstable medical illnesses. Patients or controls with contraindications to MRI were excluded. All participants also underwent a large battery of neuropsychological tests (see Kemp et al. [4] for a complete description of assessments) in addition to the MMSE [40] and to the 2 attention tasks (see below). On the basis of the MMSE scores, 70 patients were at the prodromal stage, 17 patients were at a mild stage of dementia, and 6 patients had moderate dementia.

This study was part of the larger cohort study AlphaLewyMA (http://clinicaltrials.gouv/ct2/show/NCT01876459) and was approved by the local ethics committee of East France (IV); all participants provided written informed consent for the study according to the Declaration of Helsinki.

### Assessment of attention

The Trail Making Test A (TMTA) [41] measures attention, visual screening, and processing speed. It consists of 25 circles, distributed over a sheet of paper. Circles are numbered 1–25, and the participant is asked to draw lines to connect the numbers in ascending order as quickly as possible. The completion time and the number of errors are recorded.

The Digit Symbol Substitution Test (DSST) [42] measures working memory, visuospatial processing, attention, and processing speed. It involves a key in which numbers 1–9 are each paired with a unique symbol. Below the key, numbers 1–9 are shown in random order. The participant is allowed 120 s to fill in the corresponding symbol for each number. Each correct pairing is scored 1 (maximum total raw score = 133). Raw scores can be converted to standard scores (total = 19).

### Neuroimaging study

We used VBM to investigate the neuroanatomical correlates of attention/speed processing performances in the DLB patients. To map the regions of atrophy related to the attentional deficit, we tested the correlation between the GM volume at a voxel level and the scores on both attentional tasks in the patient group. Each patient underwent a high-resolution anatomical MRI scan at inclusion. T1-weighted three-dimensional anatomical images were obtained using a 3T MRI scanner (Verio 32-channel Tim Siemens scanner; Siemens, Erlangen, Germany) using a volumetric magnetization-prepared rapid acquisition with gradient-echo (MPRAGE) sequence (FOV = 256 × 256 mm^2^, image matrix = 256 × 256, slice thickness = 1 mm, repetition time = 1900 ms, echo time = 2.52 ms, flip angle = 9°). VBM analyses included image preprocessing and statistical analyses. These steps were carried out using the SPM12 software package (Wellcome Department of Imaging Neuroscience, London; http://www.fil.ion.ucl.ac.uk/) running on Matlab R2017b (MathWorks, Natick, MA, USA). Anatomical MRI images were spatially preprocessed using standard procedures [43]. All T1-weighted structural images were first segmented, bias-corrected, and spatially normalized to the Montreal Neurological Institute (MNI) space using an extension of the unified segmentation procedure that includes six classes of tissue [44]. The DARTEL registration toolbox was then used to build a study-specific template and to bring into alignment all of the segmentation images. The VBM analysis was done on modulated GM images; that is, the GM value in each voxel was multiplied by the Jacobian determinant derived from the spatial normalization. This procedure preserves the total amount of GM from the original images. These modulated GM images were smoothed with a Gaussian kernel (FWHM, 8 mm).

### Statistical analysis

#### Behavioral analyses

Between-group differences for demographic data, MMSE scores, and attention scores on both tasks of interest (time in seconds and raw scores on the TMTA and DSST, respectively) were analyzed in STATISTICA using *t* tests for quantitative measures. For categorical measures (sex, handedness, fluctuations, hallucinations, and parkinsonism), *χ*^2^ tests were applied.

#### VBM analyses

Statistical correlations between local GM volume and scores on both attention tests were then investigated using the general linear model (GLM). Time in seconds on the TMTA and raw scores on the DSST were tested successively in the patient group. The correlations were tested using *t*-contrasts (one-tailed test), assuming that increased time in the TMTA and decreased raw scores on the DSST would be associated with decreased GM volumes. Subjects’ age, educational level, and the total GM volume were considered as covariates in the model. The results were analyzed using different statistical significance, either corrected for multiple comparisons using FWE or FDR at *p* < .05 or without correction at *p* < .001, while considering a spatial extent of 50 voxels. The XjView software package (http://www.alivelearn.net/xjview/) was used to visualize the results and to report the brain regions involved in the detected clusters. To ensure that the main results were independent of the severity of the disease, we also performed an additional analysis in considering MMSE scores as a nuisance covariate in an additional model of analysis.

## Results

### Behavioral results

Table 1 shows the demographic and group characteristics of healthy controls and patients with DLB. The groups were well matched in terms of age, educational level, sex, and handedness. Patients presented with significantly more fluctuations, hallucinations, and two out of the three features of parkinsonism (akinesia and rigidity) than controls (Table 1). As expected, significant differences were found between patients and controls on neuropsychological measures (Table 2). Behavioral analyses revealed in patients, compared to controls, significantly lower scores on the MMSE (*p* < .001) and higher scores on the DSST (*p* < .001). DLB patients also took significantly more time to achieve completion of the TMTA (*p* = .003), indicating an impairment in the speed of processing.

**Table 2:** MMSE, TMTA, and DSST scores of dementia with Lewy bodies (DLB) patients and healthy control subjects

	DLB patients	Controls	*p* value
MMSE	25.5 (3.8)	28.9 (0.9)	*p < .001*
TMTA, time in seconds, *N* = 87	76.2 (59.9)	41.0 (11.7)	*p = .0026*
DSST, raw scores, *N* = 81	39.3 (15.9)	62.4 (15.6)	*p < .001*

Standard deviations are shown in parentheses. Significant *p* values are in italics

*MMSE* Mini-Mental Status Examination, *TMTA* Trail Making Test A, *DSST* Digit Symbol Substitution Test

### Neuroimaging results

#### TMTA

**Fig. 1:**
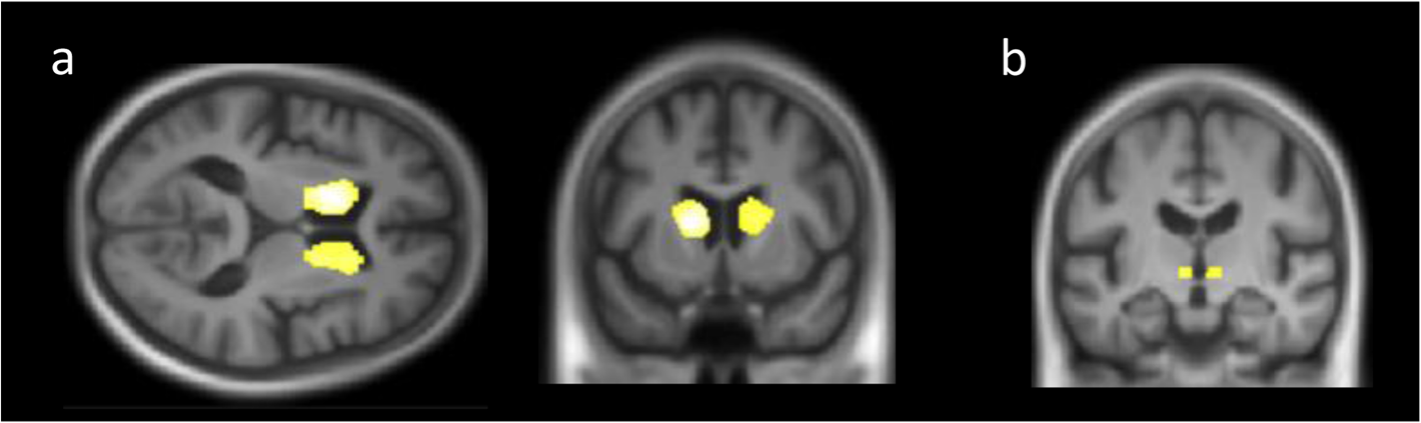
The brain regions correlated with the Trail Making Test A (TMTA) in dementia with Lewy bodies (DLB) patients. Negative correlations were found between TMTA scores and two large bilateral clusters including primarily the caudate nucleus (**a**) (*p* < .05 FDR and FWE corrected), and a third cluster involving the subthalamic nuclei (**b**) (*p* < .05 FDR corrected)

Six DLB patients were excluded from the analysis because TMTA scores were missing. VBM analyses revealed that atrophy in bilateral basal ganglia regions was associated with impaired performances on the TMTA (Fig. 1 and Table 3). More precisely, there were significant negative correlations between mean RT and GM volume in two large clusters involving bilaterally the caudate nucleus, the putamen, the thalamus, and the right globus pallidus (*p* < .05, FDR corrected); notably, the left caudate was significant at a voxel-level threshold of *p* < .05 FWE corrected (− 15 10.5 9, *t* = 5.07). Negative correlations were also found in the subthalamic nuclei (*p* < .05, FDR corrected).

**Table 3:** Brain regions negatively correlated with the time on the Trail Making Test A (TMTA; significant at *p* < .05, FDR corrected) and positively correlated with raw scores on the Digit Symbol Substitution Test (DSST; significant at *p* < .001 uncorrected) for the DLB group

Brain region	R/L	BA	*k*	*x*	*y*	*z*	*T*
TMTA (*N* = 87)							
Cluster 1							
Caudate nucleus (head and body)	L	na	1033/1646	*− 15*	*10.5*	*9*	5.07*
Putamen	L	na	121/1646	− 18	15	3	3.71
Globus pallidus	L	na	18/1646	− 13.5	4.5	3	3.6
Thalamus (ventral anterior nucleus)	L	na	21/1646	− 10	− 1.5	6	3.2
Cluster 2							
Caudate nucleus (head and body)	R	na	1000/1484	12	0	13.5	4.44
Putamen	R	na	114/1484	22.5	13.5	10.5	3.82
Cluster 3							
Subthalamic nucleus	L/R	na	230	− 4.5	− 13.5	− 3	4.1
DSST (*N* = 81)							
Cerebellum	L	na	268	− 43.5	− 70.5	− 43.5	4.1
Thalamus (ventral lateral nucleus)	L	na	201	− 15	− 16.5	10.5	3.77
Inferior frontal gyrus	R	44/45	105	55.5	18	19.5	3.59

*x*, *y*, *z* MNI coordinates of the cluster peak; *BA* Brodmann area; *k* cluster extent; *T t* value for the cluster peak

*Cluster peak significant at *p* < .05 FWE corrected

#### DSST

**Fig. 2:**
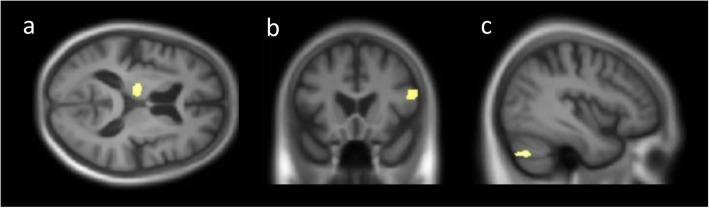
The brain regions correlated with the Digit Symbol Substitution Test (DSST) in dementia with Lewy bodies (DLB) patients (*p* < .001 uncorrected). Positive correlations were found between DSST scores and the left thalamus (**a**), the right inferior frontal gyrus (BA 45) (**b**), and the left cerebellum (**c**)

Twelve DLB patients were excluded from the analysis because DSST scores were missing. Our results revealed positive correlations between DSST raw scores and three distinct clusters located in the left cerebellum, left thalamus, and right inferior frontal gyrus (Brodmann areas [BAs] 44 and 45) at *p* < .001 uncorrected (Fig. 2). No clusters were found, either at *p* < .05 FDR corrected or at *p* < .05 FWE corrected.

#### Additional analysis

When MMSE scores as a nuisance covariate were included, there were negative correlations between mean RT on the TMTA in two large clusters involving bilaterally the caudate nucleus and the putamen, as well as the left thalamus (*p* < .0005, uncorrected). Positive correlations were found between DSST scores and a cluster located in the left cerebellum (*p* < .001, uncorrected).

## Discussion

The aim of the present study was to investigate which brain regions are involved in the attentional difficulties of DLB patients. For this purpose, we used VBM correlational analysis between behavioral performances on two typical clinical measures of attention and the degree of GM cerebral atrophy. As expected, we found an attentional deficit by means of tasks such as the TMTA and the DSST that are used in clinical routine. In accordance with our hypothesis, volumetric analyses highlighted the correlations between altered attentional scores and decreased volumes in the basal ganglia: in the striatum (mainly the caudate nucleus) and subthalamic nucleus for the TMTA, in the left thalamus for both tasks, and in the right inferior frontal gyrus (BAs 44 and 45) and the left cerebellum for the DSST. The results of our additional analysis, including MMSE scores as a nuisance covariate, confirmed that correlations between TMTA mean RT and caudate nucleus and between DSST scores and left cerebellum were not explained by the severity of the disease, reinforcing the role of these regions independently of the progression of the disease.

By comparing our group of DLB patients to healthy controls, we were able to confirm the existence of attentional deficits, even at the early stage of the disease, which is consistent with the literature [4, 6, 45]. Indeed, the majority of the patients included in the present study were at the prodromal or mild stages of the disease (only 6 out of the 93 patients were at the moderate stage at the time of the evaluations). The 2 tasks we used have in common that they measure the speed of processing as they require a series of operations to be performed under time pressure, and they both involve visual analysis, focused attention, response selection, and motor execution, yet each task has its own specificity. The DSST is a widely used, standardized psychometric test that also targets the maintenance of stimulus-response associations, and has high re-test reliability [42, 46]. Hence, this measure involves more operations than the TMTA, which might explain, based on the variability of patterns of atrophy in DLB patients, why correlations between DSST scores and the degree of atrophy are less specific of one particular brain region. Conversely, the TMTA requires more basic processes involving the correct sequencing of simple elements. The present results are in accordance with the literature suggesting that this measure constitutes a key discriminator between DLB and AD [11].

Negative correlations were found between mean reaction times in the TMTA and clusters that were very delimited to the regions of the basal ganglia, including primarily the caudate nucleus, the putamen, the globus pallidus bilaterally, the left thalamus, and the subthalamic nuclei, meaning that the slower the processing speed, the more these regions were atrophied. The basal ganglia consist of an array of the subcortical nuclei, including the caudate nucleus and the putamen (collectively referred to as the striatum in humans), the globus pallidus, the subthalamic nuclei, and the substantia nigra [47].

The striatum is involved in loops interacting with the prefrontal cortex (PFC). There are at least five of these circuits anatomically described: motor, oculomotor, dorsolateral PFC, orbital PFC, and anterior cingulate circuits [48], supported by both neuroanatomical and neuroimaging studies [47]. They all originate in the PFC and form a loop passing through the striatum (caudate or putamen), globus pallidus, and substantia nigra and finally through the thalamus. In the present study, part of the striatum, the caudate nucleus, was more involved in the dorsal part of its head, and our results also indicate that the most significant correlation was for the left caudate nucleus, for which the cluster peak reached significance at FWE-corrected level. Previous physiological, disease, and lesion studies have demonstrated that the caudate nucleus is associated with processing speed. Patients with traumatic brain injury and initial severe concussion presented a correlation between the volumes of caudate and the speed of processing, assessed using the Wechsler Adult Intelligence Scale-IV (WAIS IV) processing speed index [49]. In rhesus monkeys, a spatial visual working memory task activated bilaterally the head of the caudate nuclei [50] (see also Derauf et al. for patients with reduced caudate volumes due to prenatal methamphetamine exposure correlated to reaction times in an attention task [51] and Spies et al. for HIV patients with previous childhood psychological trauma presenting with diminished volume of the left caudate nucleus, together with slower processing speed [52]). However, in prodromal Huntington’s disease, where the caudate nucleus is atrophied, no correlation was found between the caudate nucleus and speed of processing, using DSST or TMTA [50, 53]. The strong correlation we found between the TMTA and the caudate nucleus could reflect the different processes involved in this task, with a more particular involvement of the dorsal caudate/dorsolateral PFC for the cognitive aspect of the task. Such a correlation has never previously been reported in DLB. However, our results confirm the robust link between processing speed and caudate lesion recently demonstrated in traumatic brain injury by Tate et al. [49]. In line with the idea that the caudate atrophy is involved in non-motor impairment in DLB patients, the volumetric study by Barber et al. [25] highlighted a reduction of the volume of the left caudate nucleus that was not itself correlated with parkinsonism symptoms. These data are consistent with the dorsal caudate nucleus being involved in cognitive rather than motor aspects of the TMTA [47, 54]. Based on all these data, we would suggest that in this pathological condition (DLB), the basal ganglia are the point of disruption of the cognitive loops, even if VBM-GM methods cannot inform us whether afferent pathways, efferent pathways, or both are disturbed.

We also found correlations between scores on the TMTA and atrophy in the subthalamic nuclei. This region was initially described as sustaining motor functions, because of its involvement in some motor symptoms, for example, in PD; interestingly, however, this initial view of its role has been revised in the past decades to incorporate wider cognitive functions [55].

Conversely, since the putamen is connected with the PFC supplementary motor area (SMA), and hence is implicated in motor circuits, this region might rather sustain the motor aspects of the TMTA. Accordingly, it has been proposed that the putamen contributes to movement preparation during self-initiated behavior [47].

The last subcortical region that we found to be correlated with attentional scores and common to both tasks was the left thalamus in its ventral portion. More precisely, the degree of atrophy of the ventral anterior nucleus was correlated to scores on the TMTA and those of the ventral lateral nucleus to scores on the DSST. It is interesting to note that distinct regions of the thalamus are also integrated into the cortico-subcortical circuits described before, receiving afferent projections from the striatum [48]. It has recently been suggested that two of the DLB core features, hallucinations and fluctuations, might be related to the pathological mechanisms at the level of the thalamus, by disturbing the thalamocortical circuits [56]. In DLB patients, attentional impairment is associated with cognitive fluctuations, for which thalamic dysfunction has been shown (Chabran E, Noblet V, Loureiro de Sousa P et al: Changes in gray matter volume and functional connectivity in dementia with Lewy Bodies compared to Alzheimer’s disease: implications for fluctuations, submitted). More importantly, our result corroborates the recent finding by Watson et al. [26], one of the two studies that, to date and to our knowledge, have looked for correlations between attention scores and GM volumes: using linear regression analysis centered on the thalamus, and attentional tasks of simple reaction time, choice reaction time, and vigilance, they found that atrophy in the ventral lateral nucleus was associated with impaired attentional function in DLB. Taken together, these data point towards a dysfunction of the thalamic ventral lateral nucleus underlying attentional impairment in DLB patients.

Overall, the implication of the subcortical regions is consistent with the subcortical syndrome clinical picture that is characterized by a general slowing of cognitive and motor functions, occurring as a consequence of strokes, vascular lesions, and metabolic diseases [57].

Turning now to non-subcortical regions, neither the insula nor other regions of the attentional networks described in the introduction were found to be correlated to attentional scores. Given the fact that the insula, which is part of the SN, has been shown to be involved in the detection of salient stimuli and might not be involved in the tasks we studied. As for the absence of involvement of the DAN [12, 13], the tasks we used might not have required either a redirection of attention or the recruitment of strong working memory processes.

On the contrary, we found positive correlations between DSST scores and atrophy in the right inferior frontal gyrus (BA 45) and in the left cerebellum. Regarding the former, if its left counterpart corresponds to the motor speech area of Broca, the right inferior frontal region has been associated with attentional control [14] and also with reasoning about indeterminate relations [58]. This appears consistent with our result since the specificity of the DSST is to associate unknown and insignificant symbols with numbers. We could also speculate that BA 45 in the right hemisphere serves as a premotor area for non-language codes.

Similar to other brain regions we have already discussed in the present paper, there is cumulative evidence, based on neuroimaging findings and neuropsychological research, suggesting that the cerebellum is involved in cognition in a way that is independent of motor functions [59]. Relevant to the combined atrophy we displayed in the cerebellum, thalamus, and PFC correlated to impaired performance on the DSST is the concept of “cognitive dysmetria” proposed by Andreasen et al. [60], observed in schizophrenic patients, which implicates a disruption of connectivity between these three nodes—cerebellum, thalamus, and prefrontal cortex—and produces, behaviorally, “difficulties in prioritizing, processing, coordinating, and responding to information” [60, 61]. Disturbed connectivity between these three brain regions, as reflected by our correlational results, might explain the impaired performances on the DSST in our group of DLB patients. As these results were reported at an uncorrected level of *p* < .001, these interpretations need to be treated with caution.

Overall, the positive correlations we found between raw scores on the DSST and GM volumes were less clearly delimited to one brain structure and less strong than with the TMTA, which might indicate a lower sensitivity of this test to gray matter damage of the basal ganglia. Conversely, DSST performance seems to be sensitive to white matter damage due to injury or disease [62, 63] and aging [64]. As a perspective, we will now examine the correlations between white matter morphometry as well as diffusion tensor imaging and attention scores on the same set of data. To disentangle the aspects of motor and/or cognitive speed impairment, we will explore motor speed independently, for instance, by focusing on walking speed in DLB patients.

## Conclusions

This is the first study to have examined the neural correlates of attentional dysfunction in DLB patients using voxel-based morphometry and attention tasks typically used in clinical routine. While the existing literature on the neurobiological correlates of attentional dysfunction in DLB is still unclear, we were able to demonstrate, using correlation analysis between attentional scores and GM volumes, the involvement of the striatum—particularly the left caudate nucleus—and the subthalamic nucleus, associated with increasing time on the TMTA, the involvement of the right inferior PFC and the left cerebellum, associated with diminished performances on the DSST, and the involvement of the left thalamus in both tasks. Partially confirming our hypotheses, these results reveal the involvement of the basal ganglia in processing speed and attention in DLB.

## Data Availability

The datasets analyzed during the current study are available from the corresponding author on reasonable request.
